# Evaluation of Mycotoxins in Infant Breast Milk and Infant Food, Reviewing the Literature Data

**DOI:** 10.3390/toxins13080535

**Published:** 2021-07-30

**Authors:** Marta Hernández, Ana Juan-García, Juan Carlos Moltó, Jordi Mañes, Cristina Juan

**Affiliations:** Laboratory of Food Chemistry and Toxicology, Faculty of Pharmacy, University of Valencia, Burjassot, 46100 València, Spain; herher5@alumni.uv.es (M.H.); j.c.molto@uv.es (J.C.M.); jordi.manes@uv.es (J.M.)

**Keywords:** breast milk, infant formula, infant cereals baby food, mycotoxin, estimated daily intake

## Abstract

In this review, an analysis focusing on mycotoxin determination in infant breast milk and infant food has been summarised for the last fifteen years of research focused on the intended population group of 1–9 months. The objective was to know the level of exposure of the child population to an estimated daily intake (EDI) of mycotoxins from the consumption of habitual foods. The EDI was compared with the tolerable daily intake (TDI) established by EFSA to estimate risk. In breast milk, the high prevalence and levels were for samples from Africa (Egypt and Tanzania) with aflatoxin M1 (1.9 μg/L and 10%), and Asia (Iran) with ochratoxin-A (7.3 μg/L and 100%). In infant formulas, high incidences and values were for samples with aflatoxin M1 from Burkina Faso (167 samples, 84%, 87 μg/kg). In cereal products, the highest incidence was for DON from the United States (96 samples), and the highest value was an Italian sample (0.83 μg/kg of enniatin B). In fruit products, patulin was the most detected in Italian (78) and Spanish (24) samples. The highest risk was observed in breast milk during the first month of age, the highest EDI for aflatoxin M1 was reported for Egypt (344–595 ng/kg bw/day) and ochratoxin-A for Iran (97–167ng/kg bw/day), representing a public health problem.

## 1. Introduction

Mycotoxins are substances produced by different moulds (genera *Aspergillus, Penicillium* and *Fusarium*) that can grow on food under certain conditions of humidity, temperature and can cause toxicological effects in humans and animals [1]. Contamination by a mycotoxigenic fungus usually occurs in the field, and sometimes during storage and distribution after harvest. A greater or lesser production of mycotoxins will depend on the variety of seed used, the storage conditions and environmental characteristics such as temperature, humidity and precipitation [2,3]. Their presence in food and othe products implies exposure to the consumers that in case of children it supposes a risk. Children are three times more susceptible to the toxic effects produced by mycotoxins compared to adults, because they have lower body mass, their metabolic rate is higher and they do not yet have a mature detoxification system [4]. Therefore, it is necessary to evaluate mycotoxin presence in raw materials and the level of exposure to children [5].

Feeding during the first months of a baby’s life is of the utmost importance for proper physiological and immune development. Breast milk is in many cases, the first food eaten by children at birth, and it is considered the best-adapted food for the needs of babies both for its nutritional and immune contribution [6]. Numerous studies indicate a decrease in developing diseases when children breastfed, among which a lower risk of sudden death throughout the first year of life. Breastfeeding also minimizes the appearance of gastrointestinal and respiratory infections. In the long term, they suffer less frequently from atopic dermatitis, allergy, asthma, inflammatory bowel disease, obesity and diabetes Mellitus [7].

The most common mycotoxins present in food are aflatoxins (AFs), ochratoxin A (OTA), zearalenone (ZEN), deoxynivalenol (DON), nivalenol (NIV), T-2, HT-2, patulin (PAT) and fumonisins (FBs).

AFs (AFB_1_, AFB_2_, AFG_1_ and AFG_2_) are among the most toxic mycotoxins and are immunosuppressive, mutagenic and teratogenic. AFs are produced by the fungi *Aspergillus flavus* and *Aspergillus parasiticus*. These fungi grow best in areas with tropical or subtropical climates and appear in foods such as nuts, cereals and their derivatives, usually during storage [1]. They resist the usual food treatments and the selection of products or other physical treatments allow reducing their content [1,8]. AFB_1_ is the most toxic, it is a potent hepatocarcinogen and has been classified as group 1 by the International Agency for Research on Cancer (IARC). AFM_1_ is a metabolite of AFB_1_ that can be found in milk. It is classified in group 2B as a possible human carcinogen [9].

OTA is produced by different species of fungi of the genera *Aspergillus* and *Penicillium* and it is not destroyed through the usual cooking procedures; temperatures above 250 °C applied for several minutes are required. It can be present in baby foods, cereals, coffee beans, cocoa, spices and nuts, mainly during the storage stage if the conditions are favourable; cereal-based foods for babies and young children could contain OTA. It has been classified as a possible human carcinogen (group 2B) by the IARC and the target organ is the kidney. It is teratogenic, embryotoxic, genotoxic, neurotoxic and immunosuppressive [1,9].

ZEN is produced by various species of Fusarium, which commonly grow in temperate and warm climate regions [1]. They appear mainly in corn, in addition to other cereals such as wheat, barley, sorghum and rye, and their products. ZEN is absorbed and metabolized in the human body giving rise to different metabolites (α-zearalenol and β-zearalenol) with associated estrogenic effects and hepatotoxicity, genotoxicity and immunosuppression [10,11]. An overview of in vivo studies on ZEN and its metabolites indicates that its toxicity and activity differs between animal species and sexes. In fact, ZEN causes adverse effects including a disturbance of the oestrous cycle, ovulation, conception and implantation, embryonic death, reduced fetal weight, reduced litter size and impaired neonatal survival in female immature pigs [11].

The species *F. graminearum* and *F. culmorum* of the genus *Fusarium* produce non-estrogenic toxins named trichothecenes, and among the most frequent is DON [1]. This mycotoxin can be found in wheat, barley, oats and corn and it is produced mainly before harvest [12]. Food processing can decrease the concentration of DON and its derivatives 3-Ac-DON and 15-Ac-DON. This mycotoxin causes vomiting, anorexia, gastrointestinal problems, it is immunosuppression and potential teratogenic. It is classified in group 3 by the IARC [1].

NIV also belongs to the group of trichothecenes and can be present in wheat, oats, barley and rye crops as well as their derivatives such as malt, beer, bread and cereal-based foods for babies and young children [1]. NIV causes immunotoxic and hematotoxic effects and it has not been possible to classify in relation to its carcinogenicity (Group 3) [9]. The levels of this mycotoxin in food are reduced with cleaning and selection of cereals. Regular cooking does not eliminate this mycotoxin, however, it can reduce them slightly; although it is known that high temperatures are needed for a long period to remove them.

T-2 and HT-2 toxins are produced mainly by the *F. sporotrichoides* spp, these appear both in grains and in the milling of oats and their derived by-products, as well as in bakery products. These toxins are not usually found at harvest time, although they are generated when the grain gets wet during storage due to poor conditions in the facilities [13].

Fumonisins are produced by the species *Fusarium verticillioides* of the genus *Fusarium*, the most frequent are FB_1_, FB_2_ and FB_3_, with FB_1_ being the most toxic. These mycotoxins prevail in cereals such as corn and to a lesser extent in wheat and derivatives, and are more frequent in places with hot climates and tropical areas [1]. FB_1_ is hepatotoxic and a possible carcinogen classified by IARC in group 2B and the target organs are kidneys and liver [1].

PAT is a mycotoxin produced by different species of *Penicillium*, *Aspergillus* and *Byssochlamys* fungi, its highest prevalence is in products derived from apple. It causes neurotoxic, immunotoxic, genotoxic, gastrointestinal and mutagenic effects. PAT does not accumulate in the body, but a high intake of this mycotoxin can cause gastrointestinal problems in humans.

The European Commission (EC) established maximum levels (MLs) for AFB_1_, OTA, ZEN, DON and the sum of FB_1_ and FB_2_ in processed cereal-based foods, baby foods for infants and young children at 0.100 µg/kg, 0.500 µg/kg, 20 µg/kg, 200 µg/kg and 200 µg/kg, respectively [14,15]. There are no MLs established for NIV in infant food by the EC. However, for PAT in apple juice and apple-based products, including applesauce and applesauce intended for infants and young children ML are established at 10 µg/kg [14]. Infant formulas and follow-on formulas, including infant milk and follow-on milk the limit for AFM_1_ is set at 0.25 µg/kg and for both raw milk, heat-treated milk and milk for the manufacture of dairy products 50 ng/kg [15,16].

In this work, a bibliographic review of the latest studies that have analysed mycotoxins in breast milk, infant formulas, cereal and fruit-based products, is presented to know the incidence and levels detected. With those results the exposure and risk to mycotoxins in the child population (since aged 1–9 months old) in different countries and continents have been evaluated. In consequence, the estimated daily intake (EDI) of mycotoxins through the consumption of habitual foods in the child population, according to the studies reviewed will be evaluated. Finally, a comparison of EDI with tolerable daily intake (TDI) established by EFSA will be presented [11,15,17,18,19].

## 2. Results and Discussion

Different analytical methods are used for the detection and quantification of mycotoxins in breast milk and baby food, suchas ELISA and mass spectrometry methods by liquid chromatography coupled to a fluorescence detector (HPLC-FD). The ELISA method is sensitive, cost-effective and easy to use because it has fewer sample cleaning processes than other detection methods such as HPLC. HPLC-based methods offer more accurate and robust analytical results compared to ELISA, but in turn require more expensive and sophisticated instrumentation, time-consuming sample preparation, and must be performed by trained technicians [8]. In this review, the occurrence tables include the analytical method used in the studies.

Recently, the studies of mycotoxins in babies’ foods include breast milk, infant formulas and infant products such as cereal or fruit. Depending on the levels of mycotoxins detected and the consumption in each month of age, the exposure and risk are different but it is an important parameter to consider in the assessment risk. In fact, in this review the results observed are presented according to the type of food as mentioned above.

### 2.1. Prevalence of Mycotoxins in Breast Milk and Baby Foods

#### 2.1.1. Presence of AFM_1_ and OTA in Breast Milk

The most frequent mycotoxins detected in breast milk are AFM_1_ and OTA, according to references [1]. In fact, only one article from Spanish samples detected low levels of ENs (20–101 ng/mL), ZEA (2–14 ng/mL) and NIV (53–67 ng/mL, with a negligible incidence (2, 3 and 13, respectively from 21 samples) [20].

Related to AFM1 and OTA, the concentration and occurrence of AFM_1_ in breast milk is highly variable, with different studies that present high percentages (100%) [21]. Its presence is directly related to the eating habits of lactating mothers due to the type of consumption pattern they carry out with a greater or lesser degree of susceptibility to contamination by AFM_1_. Diets composed of cereals, spices, seeds, nuts and cow’s dairy products are more likely to be contaminated by AFM_1_ [8]. Another important factor that influences the differences in data between different studies is the sensitivity of the analytical method used [2]. The available data on the prevalence and concentration of AFM_1_ in the studies are shown in Table 1, in which they have been carried out using different analytical methods (ELISA, HPLC/FD and LC-MS/MS).

Literature reports that there is a high incidence of AFM_1_ in countries such as Tanzania, Iran, Jordan, Serbia and Turkey in which the presence of this mycotoxin is present in 100% of the analyzed samples; while low prevalence is reported in Cameroon, Brazil and Italy with a frequency of AFM_1_ ranging between 2 and 5% of samples [22].

The highest level of AFM_1_ was observed in breast milk from Egypt in a range of 200–19,000 ng/L [22]. In this country, the appearance of AFM_1_ in breast milk was related to the consumption of contaminated corn oil, peanuts and raw milk [8]. Studies conducted in the United Arab Emirates, Iraq, Sudan and Ghana reported high concentration values of 210–4060 ng/L, 100–3010 ng/L, 7–2561 ng/L and 20–1816 ng/L, respectively [4,22].

The highest incidence was observed in Africa, however, there was an incidence of AFM1 in all the continents studied. The different studies carried out in Europe indicate a range of 1–570 ng/L and a mean of 54 ng/L as reported in Table 1. The highest level of AFM1 on this continent has occurred in Serbia where 100% of breast milk samples from lactating mothers had levels ranging from 5 to 570 ng/L, confirming a high exposure of newborns to this mycotoxin. In other countries such as Italy, a prevalence of 4% was reported in the 82 milk samples analyzed with the highest concentrations at 55 ng/L [22]. Another study was conducted in Ankara, Turkey, in which 75 samples of breast milk were analyzed, and a limit of detection (LOD) of 5 ng/L was established and all samples presented AFM_1_ in a range of 61–300 ng/L [23]. In Portugal, 67 breast milk samples were analyzed, 33% of them exceeded the LOD of 5 ng/L [24].

In Asia, the concentrations of AFM1 reported are also high, with ranges varying between <1 and 4060 ng/L. Here, we highlight the United Arab Emirates range of 210–4060 ng/L [4] and Iraq’s at 100–3010 ng/L [22]. In Iran, an incidence of 100% for AFM1 was found in the analyzed samples. On the contrary, the level was low, in Iran, with a range between 0.1 and 14 ng/L. The consumption of cow’s milk in rural areas of Iran reported a low concentration (5–8 ng/L). Other studies carried out in this country indicated that the main source of AFB1 was from cereals, peanut butter, vegetable oil and rice, as reflected in the levels of AFM1 in breast milk [8,22].

America is one of the continents with the lowest AFM_1_ range (<1–458 ng/L) observed in the different studies reviewed. The country with the highest concentration was Ecuador with an average of 45 ng/L in which all positive samples exceeded the ML set by EC (25 ng/L) [16,22].

Africa is the continent with the highest level of AFM_1_. Positive breast milk samples, ranged from 0.2 ng/L in Zimbabwe to 19,000 ng/L in Egypt with a mean of 7100 ng/L. A Work carried out in Ghana and Sudan indicated high concentrations of this mycotoxin [4,22]. Children who were breastfed longer on this continent had lesser exposure to AFs because the food during weaning is based on corn and cassava, which is usually contaminated with these metabolites [4]. Other foods such as beans and wheat flour were associated with AFM_1_ contamination of breast milk in Nigeria [8]. In addition to the favourable environmental conditions for the production of AFs that occur in this continent, the inadequate storage of raw material, technological obstacles, poverty and lack of knowledge, both on the part of the farmer and the consumer, are related to this high levels of AFM_1_ in countries such as Egypt, Sudan and Tanzania [8,22].

Worldwide, the AFM_1_ values ranges from 0.2–19,000 ng/L (Brazil and Egypt, respectively) and an average of 264 ng/L, exceeding the limits set by the EC (25 ng/L) [16,22]. A study on the prevalence and concentration of AFM_1_ in human breast milk based on a global systematic review and meta-analysis indicated an increase in the prevalence of AFM_1_ with increased of rainfall and poverty [8].

In recent years, few studies have been carried out worldwide on the incidence and concentration of OTA in breast milk compared to those carried out for the analysis of AFM_1_ [20]. Its presence in breast milk is related to the dietary habits of the mother, which are different depending on the country, as in AFM_1_ [4].

The data collected is reported in Table 2. It is shown that OTA incidence ranges between 4–100%, with low concentrations of OTA in samples from Egypt, Brazil and Slovakia [4,20]. The highest prevalence corresponds to Chile, Iran and Turkey, of which 100% of the analyzed samples are OTA positive. In Chile, the concentration of OTA was found between a range of 10–184 ng/L not exceeding the limit set by EC (500 ng/L), on the contrary, in Iran and Turkey the concentrations of OTA found did exceed the ML, the ranges were 2–7340 ng/L and 621–13,111 ng/L, respectively [15,22,23].

In Italy, a study of OTA performed in breast milk indicated of a prevalence of 79% with low concentrations from 1 to 75 ng/L, indicating a positive relationship between the presence of OTA and the consumption of pork, sweets, soft drinks and seed oils [22]. However, the highest concentration of OTA (13,111 ng/L) and prevalence (100%) was found in Turkey [24]. A study conducted in Norway indicated that OTA registed levels in human milk were related to the consumption of liver pate and cakes by nursing mothers.

In Egypt, a significant relationship was observed between high concentrations of OTA in breast milk and the appearance of initial kidney damage in children [4].

The continent with the highest average OTA concentration is Asia with 1007.5 ng/L and a range of 2–7340 ng/L (Iran), surpassing the ML in EC (500 ng/L) according to studies carried out in recent years. This is followed by Europe with an average of 279 ng/L and a concentration range that oscillates between 1 ng/L (Italy) and 13,111 ng/L (Turkey), one value higher than the ML established by EC (500 ng/L). In America, the mean is 54 ng/L with a range of 1 ng/L (Brazil) and 186 ng/L (Chile). In Africa the number of studies carried out to detect the level and frequency of OTA in breast milk is very scarce.

Globally, the range of OTA is between 1 ng/L (Italy) and 13,111 ng/L (Turkey), and an average of 331.4 ng/L (Table 2).

Despite the difference in data obtained in different studies at a global level, it was possible to determine a strong relationship with the frequency and concentration of OTA in human breast milk related to geographical location and especially related to eating habits, culinary style and culture. The diet of nursing mothers is determining factors in the presence of this mycotoxin [22].

#### 2.1.2. Presence of Mycotoxins in Infant Formulas

Incidence and levels of mycotoxins in infant formulas in studies from 2006 to 2019 are collected in Table 3.

The highest concentrations of mycotoxins found in infant formula samples are AFB_1_ 87,400 ng/kg with a high prevalence of 84% of the samples being positive, and OTA at levels of 3200 ng/kg, but a prevalence of 8% for both metabolites found in Africa (Burkina Faso) exceeded the limits established by Europe [22]. A study carried out in Egypt in which AFM_1_ was analyzed in 125 samples of breast milk and 125 samples of infant formulas indicated a higher mean concentration in breast milk 74 ng/kg compared to 9.79 ng/kg in infant formulas [25].

In samples from Asia in South Korea, levels of NIV were found in a range of 16,500–17,900 ng/kg and ZEN at 300–17,600 ng/kg. The occurrence of these mycotoxins was of 19 and 25%, respectively [22,28].

The main mycotoxins detected in infant formula in Europe were AFM_1_, AFB_1_, OTA and ZEN found in infant formulas. In Portugal, the presence of AFB_1_ was detected with a low prevalence of 14% and in much lower ranges compared to the concentrations found in Africa [22]. AFM_1_ was found in countries such as Spain, Italy, Portugal and Turkey at levels ranging 0.6 ng/kg (Spain) and 41 ng/kg (Portugal) [22,29,30]. The highest prevalence was in a study carried out in Portugal which showed 86% of samples were positive [19]. The prevalence of OTA in Europe was 72% in Italy, 43% in Portugal and 19% in Turkey. The highest level of OTA was 690 ng/kg in an Italian sample, which exceeded the ML set by Europe of (500 ng/kg) [15]. Another study in Italy, in which 130 samples of infant formulas were analysed, found ZEN levels that ranged between 420–760 ng/kg with a low prevalence of 8% of positive samples [31].

In America, the presence of AFM1 stands out in a study from Brazil in which the mycotoxin was prevalant in 44% of positive samples with a mean of 24 ng/kg. The highest concentration of OTA was observed in Canadian samples at 886 ng/kg [22].

#### 2.1.3. Presence of Mycotoxins in Cereal-Based Products for Infant

Cereal-based products are one of the main sources of human exposure to mycotoxins. This contamination is of great relevance for certain population groups such as children, infants and babies due to their vulnerability to toxicity induced by mycotoxins [32].

Table 4 shows data from the review of studies on the prevalence and concentration of mycotoxins in cereal-based products such as AFs, OTA, PAT, ZEN and DON, they also indicate data on mycotoxins not regulated in Europe such as NIV, BEA, STG, ENs, T-2 and HT-2 [22].

A study carried out in Tunisia revealed the presence of various mycotoxins in cereals and cereal-based products intended for children’s consumption. In general, 67% of the samples were contaminated by mycotoxins, the most frequent were DON and ENB, which were found in 63% of the 32 samples analyzed at concentrations of 11 µg/kg and 93 µg/kg, respectively. The toxins 15-AcDON, HT2 and ZEN were also found but with a lower incidence. Detected levels of DON and ZEN did not exceed the ML set in Europe for cereal-based foods in babies (200 µg/kg and 20 µg/kg), therefore no toxicological risks were recorded for child consumers in this country [14,33].

In Italy, a study was conducted in which the content of 23 different mycotoxins was analyzed in 75 baby food samples (23 cereal-based samples). Tested samples were 92% positive for OTA, DON, HT-2, FUS-X, NIV, ENB, ENB1, ENB4, ENA1 and BEA. DON was the most detected (25%) at concentrations of 268 µg/kg, 2.6% exceeded the ML of DON (200 µg/kg) set by the European legislation for processed samples of cereal-based baby food for infants, toddlers, infant formulas, and follow-on formulas [5,14].

For not regulated mycotoxins in Europe, the highest prevalence was 43% for BEA (Spain) while the highest concentration was 832 µg/kg for ENB found in a cereal sample from Italy [5,22].

AFM_1_ presented a very low prevalence in studies carried out in Portugal and Spain, 20% and 3% respectively. In Spain, the maximum concentration of AFM_1_ (250 ng/kg) and AFB_1_ (3110 ng/kg) exceeded the regulations established by the EU of 25 ng/kg and 100 ng/kg, respectively [16,22].

In a Spanish study, DON was detected in concentrations ranging between 36–245 µg/kg, the highest concentration was reported in a sample that contained rice and oats as main ingredients. Only one sample exceeded the maximum level for DON (200 µg/kg) [12,14].

A study conducted in Iran indicated the highest concentration for AFB_1_ of 15 µg/kg with a prevalence of 69% [22].

In the USA, 64 samples of infant cereals were analyzed, 78% of them contained some mycotoxin. A total of 21 samples exceeded the European Union MLs, 16 samples were greater than the OTA limit (0.5 µg/kg) [15] in ranges of 1–14 µg/kg and a sample with ranges between 0.5–32 µg/kg exceeded for ZEN (20 µg/kg) [14]. AFB_1_ was detected in 3 samples in a range of 2.4–5.9 µg/kg, AFB_2_ was in 14 (22%) in the range of 1.1–1.5 µg/kg and 9 samples (15%) were contaminated with AFG_2_ in the range of 0.7–1.7 µg/kg. DON appeared in 42 positive samples (66%), it was the most detected mycotoxin in the infant cereals analyzed in this study, with concentrations ranging between 1.4 and 147 µg/kg [34].

In another study carried out in the United States in which 147 samples of infant cereals were analyzed, T-2, OTA, ZEN, FB and DON were found. DON was the higher prevalence with 65% of positive samples in a range of 34–258 µg/kg; while had 19 samples exceeded the ML set by Europe for OTA (0.5 µg/kg) and ZEN (20 µg/kg) [14,15,22].

OTA was also detected in Canada with a prevalence of 41% of 627 samples analyzed, in Turkey in 19% of 21 samples [22] and Syria in 43% of the 30 samples analyzed [35].

#### 2.1.4. Presence of Mycotoxins in Fruit-Based Products for Infants

Data on mycotoxins in fruit-based children’s products carried out in recent years (2006–2019) are shown in Table 5 and Table 6. The incidence of mycotoxins in baby fruit purees, compote and juices is not very high. In different studies reviewed indicate that PAT was the only mycotoxin found in the majority of apple-based fruit products consumed by young children [36]. Except for a study carried out in Syria in which OTA was found in 4 fruit purees, with a prevalence of 67% and a range that oscillated between 0.019–0.156 µg/kg [35].

In Spain, the presence of PAT in 161 samples of apple juice, 77 of solid apple-based foods and 146 of apple-based baby food were analysed and among them, the frequency of PAT was 42% in applesauce, 32% in multi-fruit compote and 25% in apple juices [37]. In Italy, 120 samples of compote and fruit puree were analysed and 65% were positive (3–9 µg/kg) [38]. In another study carried out in Italy, PAT was not detected in 26 fruit juices and purees samples analysed [5].

In China, in 30 apple juice samples, PAT was detected in 19 samples with a mean of 9.3 µg/kg [39]. In Serbia, 44% of 48 infant fruit juices analyzed contained PAT (3–27 µg/kg) and 16.7% of 66 infant purees presented PAT (6% applesauce and 20% in multifruit puree) [40].

The highest prevalence of PAT in juices, purees and fruit compotes was reported in a study carried out in Qatar, in which 100% of the samples were positive for this mycotoxin. The MLs set by EC for PAT (10 µg/kg) were exceeded in samples of purees and fruit juices in Serbia and in apple juices in China [15,41].

One of the most important factors that can influence the incidence in different countries is the influence of the processing stages and the type of storage carried out in apple-based products, since the processing and washing stages of raw materials reduce the PAT content in apple juice [39].

### 2.2. Estimated Daily Intake of Mycotoxins in Infant Food Products

The daily exposure of infants and young children to mycotoxins is a big concern since their lower body weights directly impact the higher risk of toxic effects when compared to adults. Moreover, once absorbed and systemically available, the distribution of the substance may be different from that in adults owing to the age-dependent changing of body composition. On the other hand, young infants are a particularly sensitive subgroup because their metabolic capacities are not yet fully developed.

The amount of contaminated food eaten by children, and thr values of estimated daily intake (EDI) of mycotoxins, are important information to help to determine risk management strategies [39]. In this study, we calculated EDI for each mycotoxin by the following equation: [EDIm ¼ (Cm × K)/bw]; with EDIm (ng/kg bw/day) for each mycotoxin; Cm: mean of mycotoxin in analyzed samples (ng/g); K: Average consumption of the commodity (g/day); bw: bodyweight used in the population group (kg) [33]. The EDIm for children has been calculated with the levels reported in the literature and the consumption of these products according to the recommendations reported by Piccinelli et al. [42] for the infant population and Butte et al. [43] for breastfeeding, with its body weight in each period of age. The obtained values are presented according the type of food in the next sections in Tables 7–11.

#### 2.2.1. Estimated Daily Intake of Mycotoxins with Consumption of Breastfeeding

Estimating the levels of mycotoxin intake in breast milk per day is a difficult task, due to its physiological characteristics, the mother herself and the frequency of breastfeeding [23]. The European Society for Paediatric Gastroenterology, Hepatology and Nutrition (ESPGHAN) advises that exclusive breastfeeding for around six months is a desirable goal for the nutrition of infants [44].

EDI of AFM_1_ and OTA in children 1 to 9 months was estimated from the mean consumption of breast milk in exclusively breastfeed children in developed and developing countries, it ranged between 630.5 g/day and 890 g/day [43]. Mean body weight for infants 1 month and 9 months was defined as 3.8 and 8.4 kg, respectively [42].

The EDI (ng/kg bw/day) of AFM_1_ and OTA for breast milk was calculated from the mean concentration of these mycotoxins (Table 1 and Table 2) per grams of breast milk ingested per day and the kilograms of body weight considered for every month.

The EDI of AFM_1_ is shown in Table 7, ranging from 25.65 to 44.34 ng/kg bw/day. The highest EDI occurs during the first month (44.34 ng/kg bw/day) and the lowest EDI occurs from the seventh to the eighth month (25.65 ng/kg bw/day).

The results shown that Africa is the continent with the highest AFM_1_ intake (79.19–136.88 ng/kg bw/day), and Egypt stands out with the highest intake during the first month (595.16 ng/kg bw/day) [22,25].

The next continent with the highest EDI of AFM_1_ is Europe (5.16–8.92 ng/kg bw/day). The highest occurs in Serbia (31,525 ng/kg bw/day) for the child’s first month. The lowest EDI occurs in Turkey (0.82 ng/kg bw/day) during the period of the seventh to eighth months of the baby’s life [22,26,27].

The EDI of OTA in breast milk consumed by infants aged between 1 and 9 months, are shown in Table 8, they range from 26.71 ng/kg bw/day (8th month) to 46.17 ng/kg/bw/day (1st month).

The highest OTA’s EDI occurred in Asia with 167.17 ng/kg bw/day (Iran, nursling of the first month) and the lowest EDI was observed in America with 0.38 ng/kg pc/day (Brazil during the eighth month) [22]. In Europe, EDI ranges from the first to the eighth month at 46.29–26.78 ng/kg bw/day. The highest EDI occurs in Turkey (89.27 ng/kg bw/day) and the lowest in this continent occurs in Italy (1.92 ng/kg bw/day) [21,22] In Africa, the scarcity of studies makes it difficult to calculate the EDI of OTA through breast milk, in Egypt the EDI ranges between 3.49–2.02 ng/kg bw/day [4].

#### 2.2.2. Estimated Daily Intake of Mycotoxin with Consumption of Infant Formula

The EDI of detected mycotoxins (AFM_1_, OTA, AFB_1_ and ZEN) in infant formulas are shown in Table 9. The highest intakes through this food are for AFB_1_ in Burkina Faso (85.55 ng/kg bw/day) and NIV in South Korea during the third month of age (72.04 ng/kg bw/day) [22,28].

The highest AFM_1_´s EDI occurs in Africa (Egypt) during the third month (2.20 ng/kg bw/day), its lowest value was indicated in Europe (Spain) during the ninth month (0.03 ng/kg bw/day) [25,29].

The highest intake of OTA through infant formula occurs in samples from Canada, especially the first months with an EDI of 3.26 ng/kg/day [22].

The EDI of ZEN varies from 0.59 ng/kg bw/day during the third month to 0.28 ng/kg bw/day of the ninth month in Italy [31].

#### 2.2.3. Estimated Daily Intake of Mycotoxin with Consumption of Cereal-Based Children’s Products

EFSA suggests introducing complementary feeding to babies between 4 and 6 months of age, so that and the ESPGHAN Committee notes that gastrointestinal and kidneys are mature enough around 4 months, so complementary feeding can be entered from The 17th week (beginning of 5th month). In this review, the EDI has been calculated for babies who introduced complementary feeding at 4–5 months of age.

EDI of mycotoxin has calculated through the consumption of cereal-based products and was carried out from the average level recorded in Table 4 and the daily consumption reported by Piccinelli et al. [42] of cereal-based infant products. The body weight used for infants was calculated firstly from the average of the 50th percentile at months for both females and males, according to the Multicentre Growth Reference Study Group, World Health Organization (WHO) [45]; and secondly by calculating the average between the values obtained in females and males. Estimated body weight for children between 5 and 9 months (6.95–8.4kg) [42]. The EDI of mycotoxins in cereals is shown in Table 10.

The highest EDI is recorded in Italy for babies at sixth month age for FUS-X (653.36 ng/kg bw/day) [5]. Regarding legislated mycotoxins, the highest EDI was observed for DON in Spain (521.76 ng/kg bw/day) and Italy (451.74 ng/kg bw/day) during the sixth month of the baby’s age [5,22].

In Africa, the highest EDI was registered for ENB and DON from the fifth to the sixth month, both are in the same ranges (51.8–133.78 ng/kg pc/day) [22,32]. In Canada (American continent), the highest EDI for OTA occurs in cereal-based products during the fifth to sixth month (2.63 ng/kg bw/day).

In Asia the highest intake of AFB_1_ occurs in Iranian samples in the previous months from the fifth to the sixth months (11.59 ng/kg bw/day) [22].

#### 2.2.4. Estimated Daily Intake of Mycotoxin with Consumption of Children’s Fruit Products

The EDI of mycotoxin through the consumption of children’s products with fruit are shown in Table 11. This was calculated from the mean concentration (ng/kg) recorded in Table 5. The highest EDIs of PAT was in apple juice from China (143.44 ng/kg bw/day) and OTA with peach and apple puree from Syria (1.53 ng/kg bw/day) this intake occur during the sixth to seventh months of the child’s age [34,39]. The EDI is higher in juices than in puree and fruit compotes especially apple (Table 11).

### 2.3. Evaluation of the Risk in Children of Exposure to Mycotoxins through Infant Feeding

Health risk assessment was performed taking into account current reference values including the verification introduced recently by EFSA and JECFA [46,47,48,49,50].

The JECFA recommended that compounds that are both genotoxic and carcinogenic should be reduced to As Low As Reasonably Achievable (ALARA) [19], and IARC has concluded that aflatoxins are carcinogenic to humans with a role in aetiology in liver cancer. Such as ALARA is a limited value, JECFA considered that the margin of exposure (MOE) approach is the preferred option to risk assessment [51]. To obtain MOE, it is recommended to use the BMD (benchmark dose), the dose that causes a low but measurable response or BMDL_10_ (benchmark dose lower confidence limit 10%), which estimates the lowest dose that is 95% certain to cause no more than 10% cancer incidence (BMDL_10_ of 0.00025 mg/kg bw/day) [19,52]. The CONTAM Panel selected the BMDL_10_ of 0.4 µg/kg bw/day for the induction of hepatocellular carcinoma by AFB1 in male rats as a reference point for the risk characterization of aflatoxins. MOEs were calculated by dividing the reference point (BMDL10), by the estimated human intakes. The Scientific Committee of the EFSA declares that a MOE of 10,000 or more for genotoxic and carcinogenic substances has a low level of risk for public health. EFSA´s proposal approach considers one specific carcinoma value of BMDL10 and only for one rat specie; however, there are many variabilities excluded such as the compound type, population group, scenarios of exposure, etc. Furthermore, the international scientific consensus is being defined and does not cover the objective of our review; therefore, this calculus has not been included.

The EDI has been compared with a tolerable daily intake (TDI) set by the EFSA [17] and the Joint FAO/WHO Expert Committee on Food Additive [48]. The highest values were observed for OTA in breast milk in samples from Iran and Turkey. The risk of OTA through the consumption of breast milk is shown in the Table 12. The highest values were between 691–1194% in Iran during the eighth and first month of the child’s age respectively, followed by Turkey 369–638%. These countries being the ones with the highest exposure and risk to the toxic effect of OTA to which children are exposed [17,21]. In the rest of the countries, the EDI in relation to the TDI set by international organism (TDI%) represented in Chile 54–94%, Egypt 14–25% and Italy 14–24% and the one with the lowest risk occurs in Brazil in a proportion that ranges between 3–5% [4,20,21].

The TDI% in infant formulas are indicated in Table 13. In this food, the values were below 23%. The highest percentage of OTA occurs in Canada, during the second to the third month of the child’s age (23.32%) in relation to TDI (14 ng/kg). This is followed by Burkina Faso in Africa (7.68–16.08%), with the lowest risks observed in Italy (6.76–14.15%) and Turkey (3.22%) [18,21]. The smallest values were for NIV and ZEN. NIV was between 2.87% (eighth to the ninth month) and 6% (second to the third month of age) in relation to the TDI (1200 ng/kg) in South Korea [18,28]. In Italy, the ratio of EDI to ZEN with respect to TDI (250 ng/kg) oscillates between 0.23% during the second to third month (this period being when the baby is more exposed) and 0.11% during the ninth month (when the lowest ratio is observed) [31,46].

In Table 14, we show the mycotoxin risk ratio (%) in cereal-based products. The percentage of OTA in relation to the TDI is the highest observed in Canada (18.79%) during the period from the fifth to the sixth month. In Africa the probability of FB_1_ and FB_2_ is very low, oscillating between 0.16–0.45% in relation to the TDI (2000 ng/kg) [15,21]. DON is one of the mycotoxins with the highest percentage in relation to the TDI in relation to the TDI (1000 ng/kg) and stands out at 52.18% in Spain during the sixth month of age of babies after the consumption of cereal-based foods [12,15]. In other countries the DON ratio ranges between 5.18–13.38% in Tunisia and 17.72–45.75% in Italy [22,33]. In Portugal the EDI in relation to the TDI of PAT (400 ng/kg) ranges between 1.01-2.6% [15,21]. In Italy, the proportion of the HT-2 toxin during the sixth month is 56.41% and stands out compared to the TDI (100 ng/kg) [5,13,49].

The TDI% in children’s products made with fruit was calculated as detected mycotoxins of OTA and PAT (Table 15). The OTA TDI% in peach and apple puree in Syria ranges between 7.17% during the sixth month and 10.91% in the seventh month, in fruit cocktail puree it varies between 6.3–9.59% during the same months [35]. The highest risk for PAT in Spain due to the consumption of applesauce is at 28.53% during the seventh month. Furthermore, in multi-fruit compote, the TDI% of PAT ranged between 17.48–26.61%. Fruit purees with the highest proportions are produced in Italy with 24.22% and the lowest in Serbia with 7.35% [38].

In juices, the highest risk is observed in China with 35.86% during the seventh month and the lowest is in Qatar with 7.77% during the sixth month of the child’s age [39,41,53].

In comparison, the TDI% of PAT is a greater proportion in apple-based liquid products than in solid foods such as compotes and purees. Children.

## 3. Conclusions

The highest incidence of mycotoxins has been observed in breast milk from Tanzania, Iran, Jordan, Serbia and Turkey where 100% of the analyzed samples contained AFM_1_. Samples from Africa showed the highest AFM_1_ values: between 0.2 ng/kg (Zimbabwe) and 1900 ng/kg (Egypt). OTA had a higher prevalence and concentration in samples from Iran (2–7640 ng/kg) and Turkey (621–13,111 ng/kg). Values that exceeded the ML set in the EU, included those from AFM_1_ in all breast milk samples from Africa, Ecuador, United Arab Emirates, Iraq, Iran, Jordan, Cyprus, Italy, Serbia and Turkey. There were also samples that exceeded the MLs for OTA in breast milk from Iran and Turkey.

In infant formulas and fruit-based products, the LM amounts of AFB_1_ (Burkina Faso) and PAT (Serbia and China), respectively, were also exceeded.

Subsequently, the highest EDI for mycotoxin occurs in breast milk, with 595.16 ng/kg bw/day of AFM_1_ in samples from Egypt, followed by OTA in samples from Iran (167.17 ng/kg bw/day). In infant formulas and fruit-based products, EDI values were lower, observing AFB_1_ intakes of 85.55 ng/kg bw/day in Burkina Faso and 143.44 ng/kg bw/day in fruit juices in China.

The risk assessment with the observed values indicates that there is a high risk of OTA for the child population of Iran (1194%) followed by Turkey (637%). These high values are usually samples from traditional cultivation and street markets, so there is a bias in the sampling, despite this, everything leads to the assumption that in any case they will be values much higher than those detected in European countries.

Given the results obtained, it is clear that the control and analysis of mycotoxins in the samples are necessary to know if prevention measures and good agricultural handling practices are implemented, which are the first tool to avoid the appearance of these mycotoxins and reduce exposure to the population.

## 4. Materials and Methods

A systematic literature review was completed using the databases Web of Science, PubMed and Scopus. We included articles that studied the incidence, prevalence and level of mycotoxins contained in samples of breast milk, infant formulas and products based on cereals and fruit commonly consumed in children aged 1 and 9 months of age, in different countries. The time frame was the last fifteen years. Eighteen articles, which met the criteria to be included in the study were analyzed and classified. To facilitate data presentation, the results were divided into four groups of baby foods types: breast milk, infant formulas and infant products based on cereals and infant products based on fruit. The information was double-checked to select bibliographies of relevant literature and summarize the information about, analytical methodology, incidence, range and mean of concentration levels of mycotoxins. Finally, the data available were used to estimate dietary exposure dietary to mycotoxins.

## Figures and Tables

**Table 1:** Amount of AFM_1_ in breast milk in different continents and countries.

Origin	N	Positives (>LOD)	Occurrence (%)	LOD (ng/L)	Range (ng/L)	Mean (ng/L)	Analitycal Method	Reference
**Africa**								
Cameroon	62	3	5	nr	5–62	nr	HPLC/FD	[22]
Egypt	150	98	65	nr	200–19,000	7100	ELISA	
Egypt	125	87	70	nr	7–329	74	ELISA	[25]
Ghana	264	59	22	nr	20–1816	nr	HPLC/FD	[4]
Kenya	204	129	63	nr	1–153	5	ELISA/HPLC-FD	[22]
Nigeria	310	171	55	10	<LOD–601	61.5	HPLC/FD	
Sudan	94	51	54	13	<LOD–2561	401	HPLC/FD	
Tanzania	143	143	100	5	10–550	70	HPLC/FD	
Zimbabwe	54	6	11	nr	0.2–50	nr	ELISA	[4]
**Total**					0.2–1900	1110		
**America**								
Brazil	310	0	0	6.25	0	0	LM-MS-MS	[22]
	194	7	4	4	13–25	9	HPLC/FD	
Colombia	50	45	90	0.6	1–19	5	HPLC/FD	
Ecuador	78	67	86	33	53–458	45	HPLC/FD	[22]
Mexico	112	100	89	nr	3–34	12	ELISA	[22]
**Total**					<1–458	16		
**Asia**								
Arab Emirates	201	107	53	nr	210–4060	nr	HPLC/FD	[4]
Iraq	20	16	80	nr	100–3010	nr	TLC	[22]
	734	369	50	nr	<1–27	6	ELISA	
	250	39	16	2.3	11–40	5	HPLC/FD	
Iran	88	88	100	nr	0.1–14	3	ELISA	[26]
Jordan	80	80	100	nr	10–137	68	ELISA	[22]
Lebanon	111	104	94	nr	<1–8	4.5	ELISA	
Malaysia	102	0	0	13	nr	nr	HPLC/FD	
Pakistan	125	94	75	nr	<1	nr	ELISA	
**Total**					<1–4060	11		
**Europe**								
Cyprus	50	40	80	5	5–28	8	ELISA	[22]
Italy	82	4	5	3	7–140	55	HPLC/FD	
Portugal	67	22	33	5	5.1–10.6	7	ELISA	[24]
Serbia	70	33	47	2	5–570	190	ELISA	
Turkey	61	8	13	5	5.1–6.9	6	HPLC/FD	[27]
Turkey	75	75	100	5	61–300	nr	HPLC/FD	[23]
Turkey	217	93	43	nr	1–80	9	ELISA	[22]
**Total**					1–570	54		
**TOTAL**					<1–19,000	264		

nr: Not reported. LOD: limit of detection HPLC/FC: high performance liquid chromatography with fluorescent detection. ELISA: enzyme-linked immunosorbent assay. LC-MS/MS: liquid chromatography mass spectrometry. TLC: thin layer chromatography.

**Table 2:** Amount of OTA in breast milk in different continents and countries.

Origin	N	Positives (>LOD)	Occurrence (%)	LOD (ng/L)	Range (ng/L)	Mean (ng/L)	Analitycal Method	Reference
**Africa**								
Egypt	120	43	36	nr	5.1–45	21.6	HPLC/FD	[4]
Sierra Leone	113	40	35	200	200–337	nr	HPLC/FD	
**Total**					5.1–337	21.6		
**America**								
Brazil	224	0	0	30	nr	nr	LC-MS/MS	[22]
Brazil	100	66	66	1	1–21	4	HPLC/FD	
Chile	11	11	100	10	44–184	106	HPLC/FD	
Chile	50	40	80	10	10–186	52	LC-MS	
**Total**					1–186	54		
**Asia**								
Iran	136	5	4	nr	5–16	nr	HPLC/FD	[22]
	171	168	98	nr	2–7340	1007	ELISA	
**Total**					2–7340	1007		
**Europe**								
Slovakia	76	23	30	5	<LOD–60	nr	HPLC/FD	[4]
Italy	82	61	74	2	5–405	30	HPLC/FD	[22]
Italy	57	45	79	1	1–75	10	HPLC/FD	[20]
Turkey	75	75	100	10	621–13,111	nr	HPLC/FD	[23]
Turkey	160	124	77	nr	761–1724	538	ELISA	[22]
**Total**					1–13,111	279		
**TOTAL**					1–13,111	331		

nr: Not reported. LOD: limit of detection. HPLC/FC: high performance liquid chromatography with fluorescent detection. ELISA: enzyme-linked immunosorbent assay. LC-MS/MS: liquid chromatography mass spectrometry.

**Table 3:** Amount of mycotoxins in infant formulas in different continents and countries.

Mycotoxin (ML, ng/kg)	Origin	N	Positives (>LOD)	Occurrence (%)	LOD (ng/kg)	Range (ng/kg)	Mean (ng/kg)	Analitycal Method	Reference
**AFB_1_** **(100)**	**Africa**								
	Burkina Faso	199	167	84	300	<LOD–87,400	3800	HPLC	[22]
	**Europe**								
	Portugal	7	1	14	1	<LOD–3	nr	HPLC	[22]
	**TOTAL**					**<LOD–87,400**	**3800**		
**AFM_1_** **(25)**	**Africa**								
	Egypt	125	54	43	<50	<LOD–21.8	9.8	ELISA	[25]
	**America**								
	Brazil	16	7	44	3	<LOD–46	24	HPLC	[22]
	**Europe**								
	Spain	69	26	38	1.8	<LOD–11.6	3.1	HPLC	[29]
	Italy	185	2	1	3	<LOD–15	14	HPLC	[22]
	Italy	13	0	0	15	nr	0	LC-MS/MS	
	Portugal	7	6	86	4	<LOD–410	nr	HPLC	
	Turkey	62	5	8	5	<LOD–22	18	HPLC	
	Turkey	34	1	9	5000	nr	6.1	ELISA	[30]
	**Total Europe**					0.6–41	8.2		
	**TOTAL**					**0.3–41**	**10.71**		
**NIV** **(nl)**	**Asia**								
	South Korea	16	3	19	4400	16,500–17,900	3200	UHPLC/UV	[28]
**OTA** **(500)**	**Africa**								
	Burkina Faso	199	15	8	50	<LOD–3200	100	HPLC	[22]
	**America**								
	Canada	416	57	14	40	<LOD–886	145	LC-MS/MS	[22]
	**Europe**								
	Italy	185	133	72	1	<LOD–690	88	HPLC	[22]
	Portugal	7	3	43	9	<LOD–136	nr	HPLC	
	Turkey	62	12	19	6	<LOD–184	20	HPLC	
	**Total Europe**					<LOD–690	54		
	**TOTAL**					**<LOD–3200**	**88**		
**ZEN** **(20000)**	**Asia**								
	South Korea	36	9	25	2500	3300–17,600	nr	UHPLC	[22]
	**Europe**								
	Italy	130	11	8	760	420–760	26	HPLC	[31]
	**TOTAL**					**420–17,600**	**26**		

nr: Not reported. nl: not legislate by Europe. ML: maximum level. LOD: limit of detection. HPLC: high performance liquid chromatography. ELISA: enzyme-linked immunosorbent assay. LC-MS/MS: liquid chromatography mass spectrometry. UHPLC: ultra high performance liquid chromatography.

**Table 4:** Amount of mycotoxins in cereal-based products in different continents and countries.

Origin	Mycotoxin	N	Positives (>LOD)	Occurrence (%)	LOD (µg/kg)	Range (µg/kg)	Mean (µg/kg)	ML (µg/kg)	Analitycal Method	Reference
**Africa**										
Morocco	FB_1_	20	1	5	0.1	<LOD–2	2	200	LC-MS/MS	[22]
	FB_2_	20	1	5	0.1	<LOD–2.3	1.8	200	LC-MS/MS GC-MS/MS	
Tusiana	DON	32	20	63	1.6	5–110	30	200	LC-MS/MS GC-MS/MS	[33]
	15-ADON	32	3	9	3.1	9–20	1	200		
	HT-2	32	1	3	0.0178	149–209	1	nl		
	ZEN	32	11	34	8.8	<LOD–44	1	20		
	ENB	32	20	63	3	6–93	30	nl		
**America**										
Canada	OTA	627	260	41	0.040	<LOD–4.8	0.59	500	LC-MS	[22]
United States	OTA	64	19	30	0.1	1–14.4	nr	500	LC-MS/MS	[34]
	DON	64	42	66	0.1	1.4–147	nr	200		
	AFB_1_	64	3	5	0.025	2.4–5.9	nr	100		
	AFB_2_	64	14	22	0.010	1.1–1.5	nr	nl		
	AFG_1_	64	0	0	0.010	nr	nr	nl		
	AFG_2_	64	9	14	0.010	0.7–1.7	nr	nl		
	FB_1_	64	1	2	0.5	<LOD–6.2	nr	200		
	FB_2_	64	5	8	0.125	<LOD–15.8	nr	200		
	HT-2	64	6	9	0.1	2.4–9.6	nr	nl		
	T-2	64	18	28	0.01	0.4–3.6	nr	nl		
	ZEN	64	33	52	NR	0.5–32	nr	20		
United States	DON	147	96	65	1.1	34–258	nr	200	LC-MS/MS	[22]
	T-2	147	3	2	0.5	<LOD–1.6	nr	nl		
	OTA	147	1	1	0.2	2	nr	0.5		
	ZEN	147	18	12	2.7	8.9–26	nr	20		
	FB	147	1	1	2.3	336	nr	200		
**Asia**										
Iran	AFB_1_	48	33	69	0.008	0.025–15.2	2.6	0.1	HPLC	[22]
Syria	OTA	30	13	43	0.038	2–329	0.09	0.5	HPLC	[35]
**Europe**										
Italy	OTA	75	15	20	0.050	<LOD–0.120	0.06	0.5	LC-MS/MS	[5]
	NIV	75	3	4	5.5	<LOD–235	19.9	nl		
	FUS-X	75	18	24	5.5	<LOD–604	146.5	nl		
	DON	75	19	25	1	<LOD–268	102.6	200		
	HT-2	75	2	3	2	<LOD–151	12.6	nl		
	β-ZEL	75	5	7	1.5	<LOD–23.2	2.5	nl		
	ENB	75	10	13	2	<LOD–832	101.3	nl		
	ENB_1_	75	1	1	5	<LOD–117	7.8	nl		
	ENB_4_	75	4	5	5	<LOD–311	38.1	nl		
	ENA_1_	75	3	4	5	<LOD–125	6.6	nl		
	BEA	75	1	1	5	<LOD–21.3	1.2	nl		
Portugal	AFM_1_	20	4	20	0.004	<LOD–0.023	nr	0.025	HPLC	[22]
	AFB_1_	20	6	30	0.001	<LOD–0.009	nr	0.1		
	OTA	20	13	65	0.009	<LOD–0.212	nr	0.5		
Portugal	PAT	20	15	75	0.9	<LOD–4.5	2.3	10	HPLC	
	OTA	20	10	50	0.006	<LOD–0.263	0.06	0.5		
Spain	AFB_1_	91	42	46	0.003	<LOD–3.11	0.09	0.1	HPLC	
	AFB_2_	91	36	40	0.002	<LOD–0.410	0.01	nl		
	AFG_1_	91	31	34	0.002	<LOD–0.420	0.02	nl		
	AFG_2_	91	10	11	0.002	<LOD–0.070	0.004	nl		
Spain	NIV	35	2	6	0.035	<LOD–100	nr	nl	LC-MS/MS	
	DON	35	9	26	0.020	70–210	nr	200		
	OTA	35	2	6	0.1	0.350–0.500	nr	0.5		
	FB_1_	35	3	9	0.022	75–100	nr	nl		
	FB_2_	35	1	3	0.025	0.075	nr	nl		
	AFG_2_	35	1	3	0.450	1.2	nr	nl		
	ZEN	35	2	6	2	10–15	nr	20		
	BEA	35	15	43	0.5	50–100	nr	nl		
	AFM_1_	35	1	3	0.08	<LOD–0.250	nr	0.025		
	STG	35	2	6	2.5	10–50	nr	nl		
Spain	DON	60	12	20	33	36–245	117	200	HPLC	[12]
Turkey	OTA	21	4	19	0.05	<LOD–0.200	0.140	0.5	HPLC	[22]

nr: not reported. nl: not legislate in Europe. LOD: limit of detection. HPLC: high performance liquid chromatography. ELISA: enzyme-linked immunosorbent assay. LC-MS/MS: liquid chromatography mass spectrometry. GC-MS/MS: gas chromatography mass spectrometry. UHPLC: ultra high performance liquid chromatography.

**Table 5:** Amount of PAT and OTA in solid fruit-based products in different continents and countries.

Mycotoxin	Origin	Food	N	Positive (>LOD)	Occurrence (%)	LOD (µg/kg)	Range (µg/kg)	Mean (µg/kg)	ML (µg/kg)	Analitycal Method	Reference
**OTA**	**Asia**								0.5		
	Syria	Peach and apple puree	3	2	67	0.050	0.079–0.120	0.099		HPLC	[35]
		Fruit cocktail puree	3	2	67	0.050	<LOD–0.156	0.087		HPLC	
	**Total** **Asia**						**0.019–0.156**	**0.093**			
**PAT**	**Asia**								10		
	Qatar	Apple compote	7	7	100	1	1.02–2.46	1.09		LC-MS/MS	[41]
	**Europe**										
	Spain	Apple compote	36	15	42	2.08	nr	7.4		HPLC	[37]
		Multifruit compote	76	24	32	2.08	nr	6.9		HPLC	
	Italy	Puree and fruit compote	120	78	65	1	3–9	6.3		HPLC	[38]
	Portugal	Apple puree	76	5	7	1.2	<LOD–5.7	NR		HPLC	[36]
	Serbia	Apple puree	16	1	6	2.9	<LOD–15	2.9		HPLC	[40]
		Multi-fruit puree	50	10	20	7.7	2–40	3.5		HPLC	
	**Total** **Europe**						1–40	5.4			
	**Total** **PAT**						**1–40**	**4.7**			

nr: Not reported. LOD: limit of detection. HPLC: high performance liquid chromatography. LC-MS/MS: liquid chromatography mass spectrometry.

**Table 6:** Amount of PAT in liquid fruit-based products in different continents and countries.

Origin	Food	N	Positives (>LOD)	Occurrence (%)	LOD (µg/kg)	Range (µg/kg)	Mean (µg/kg)	ML (µg/kg)	Analitycal Method	Reference
**Asia**								10		
China	Apple juice	30	19	63	1200	<LOD–37.3	9.3		HPLC	[39]
Qatar	Apple juice	6	6	100	1000	6.13–7.7	3.1		LC-MS/MS	[41]
**Total Asia**						<LOD–37.3	6.2			
**Europe**										
Spain	Apple juice	12	3	25	2080	nr	7.5		HPLC	[37]
Italy	Fruit juice and puree	26	0	0	15	nr	nr		LC-MS/MS	[5]
Serbia	Fruit juice	48	21	44	8300	3–27	3.6		HPLC	[40]
**Total Europe**						3–27	5.5			
**TOTAL**						**<LOD–67.3**	**19.5**			

nr: Not reported. LOD: limit of detection. HPLC: high performance liquid chromatography. LC-MS/MS: liquid chromatography mass spectrometry.

**Table 7:** Estimated daily intake of AFM_1_ from breast milk consumption.

**EDI (ng/kg bw/day)**										**Reference**
**Age (months)**	0–1	1–2	2–3	3–4	4–5	5–6	6–7	7–8	8–9	
**Weight (kg)**	3.8	4.85	5.75	6.4	6.95	7.4	7.78	8.1	8.4	
Consumption * (g/day)	630.5	682.5	666.5	774	787	829	803.5	777.5	890	
**Origin**										
**Africa**										
Egypt	595.16	504.77	417.23	433.80	406.18	401.84	370.46	344.31	380.05	[25]
Kenya	0.87	0.74	0.61	0.63	0.59	0.59	0.54	0.50	0.56	[22]
Nigeria	10.20	8.65	7.15	7.44	6.96	6.89	6.35	5.90	6.52	
Sudan	66.53	56.43	46.64	48.50	45.41	44.92	41.41	38.49	42.49	
Tanzania	11.61	9.85	8.14	8.47	7.93	7.84	7.23	6.72	7.42	
**Total Africa**	136.88	116.09	95.96	99.77	93.42	92.42	85.20	79.19	87.41	
**America**										
Brazil	1.58	1.34	1.11	1.15	1.08	1.06	0.98	0.91	1.01	[22]
Colombia	0.83	0.70	0.58	0.60	0.57	0.56	0.52	0.48	0.53	
Ecuador	7.47	6.33	5.23	5.44	5.10	5.04	4.65	4.32	4.77	[22]
Mexico	1.99	1.69	1.40	1.45	1.36	1.34	1.24	1.15	1.27	[22]
**Total America**	2.97	2.52	2.08	2.16	2.02	2.00	1.85	1.72	1.89	
Asia										
Iran	0.87	0.74	0.61	0.63	0.59	0.59	0.54	0.50	0.56	[26]
Jordan	11.28	9.57	7.91	8.22	7.70	7.62	7.02	6.53	7.20	[22]
Lebanon	0.75	0.63	0.52	0.54	0.51	0.50	0.46	0.43	0.48	
**Total Asia**	4.30	3.65	3.01	3.13	2.93	2.90	2.68	2.49	2.75	
**Europe**										
Cyprus	1.33	1.13	0.93	0.97	0.91	0.90	0.83	0.77	0.85	[22]
Italy	9.13	7.74	6.40	6.65	6.23	6.16	5.68	5.28	5.83	
Portugal	1.23	1.04	0.86	0.89	0.84	0.83	0.76	0.71	0.78	[24]
Serbia	31.53	26.74	22.10	22.98	21.52	21.29	19.62	18.24	20.13	
Turkey	1.41	1.20	0.99	1.03	0.96	0.95	0.88	0.82	0.90	[27]
**Total Europe**	8.92	7.57	6.26	6.50	6.09	6.02	5.55	5.16	5.70	
**Total**	**44.34**	**37.60**	**31.08**	**32.32**	**30.26**	**29.94**	**27.60**	**25.65**	**28.31**	

* Average consumption of breast milk [43].

**Table 8:** Estimated daily intake of OTA through the consumption of breast milk.

**EDI (ng/kg bw/day)**										**Reference**
**Age (months)**	0–1	1–2	2–3	3–4	4–5	5–6	6–7	7–8	8–9	
**Weight (kg)**	3.8	4.85	5.75	6.4	6.95	7.4	7.78	8.1	8.4	
Consumption * (g/day)	630.5	682.5	666.5	774	787	829	803.5	777.5	890	
**Origin**										
**Africa**										
Egypt	3.49	2.96	2.45	2.55	2.38	2.36	2.18	2.02	2.23	[4]
**America**										
Brazil	0.66	0.56	0.47	0.48	0.45	0.45	0.41	0.38	0.42	[22]
Chile	13.11	11.12	9.19	9.55	8.95	8.85	8.16	7.58	8.37	
**Total America**	6.89	5.84	4.83	5.02	4.70	4.65	4.29	3.98	4.40	
**Asia**										
Iran	167.17	141.78	117.19	121.84	114.09	112.87	104.05	96.71	106.75	[22]
**Europe**										
Italy	3.32	2.81	2.33	2.42	2.26	2.24	2.07	1.92	2.12	[20]
Turkey	89.27	75.71	62.58	65.06	60.92	60.27	55.56	51.64	57.00	[22]
**Total Europe**	46.29	39.26	32.45	33.74	31.59	31.26	28.81	26.78	29.56	
**Total**	**46.17**	**39.16**	**32.37**	**33.65**	**31.51**	**31.17**	**28.74**	**26.71**	**29.48**	

* Average consumption of breast milk [43].

**Table 9:** Estimated daily intake of mycotoxins through the consumption of infant formulas.

**Mycotoxin**	**EDI (ng/kg bw/day)**										**Reference**
	**Age (months)**	0–1	1–2	2–3	3–4	4–5	5–6	6–7	7–8	8–9	
	**Weight (kg)**	3.8	4.85	5.75	6.4	6.95	7.4	7.78	8.1	8.4	
	Consumption * (g/day)	81.7	107.5	129	129	150.5	150.5	120.4	120.4	90.3	
	**Origin**										
**AFB_1_**	**Africa**										
	Burkina Faso	81.70	84.23	85.55	76.59	82.29	77.28	58.81	56.48	40.85	[22]
**AFM_1_**	**Africa**										
	Egypt	2.10	2.17	2.20	1.97	2.12	1.99	1.52	1.46	1.05	[25]
	**America**										
	Brazil	0.52	0.53	0.54	0.48	0.52	0.49	0.37	0.36	0.26	[22]
	**Europe**										
	Spain	0.07	0.07	0.07	0.06	0.07	0.06	0.05	0.05	0.03	[29]
	Italy	0.03	0.31	0.32	0.28	0.30	0.28	0.22	0.21	0.15	[22]
	Turkey	0.26	0.27	0.27	0.24	0.26	0.24	0.24	0.18	0.13	[30]
**NIV**	**Asia**										
	South Korea	68.80	70.93	72.04	64.50	69.29	65.08	49.52	47.57	34.04	[28]
**OTA**	**Africa**										
	Burkina Faso	2.15	2.22	2.25	2.02	2.17	2.03	1.55	1.49	1.08	[22]
	**America**										
	Canada	3.12	3.21	3.26	2.92	3.14	2.95	2.24	2.16	1.56	[22]
	**Europe**										
	Italy	1.89	1.95	1.98	1.77	1.91	1.79	1.36	1.31	0.95	[22]
	Turkey	0.43	0.44	0.45	0.40	0.43	0.41	0.31	0.30	0.22	
**ZEN**	**Europe**										
	Italy	0.56	0.58	0.59	0.52	0.56	0.53	0.40	0.39	0.28	[31]

* Average consumption of infant formulas [43].

**Table 10:** Estimated daily intake of mycotoxins through consumption of cereal-based products.

**Origin**	**EDI (ng/kg bw/day)**						**Reference**
	**Age (months)**	4–5	5–6	6–7	7–8	8–9	
	**Weight (kg)**	6.95	7.4	7.78	8.1	8.4	
	Consumption * (g/day)	12	33	27	26	22	
	**Mycotoxin**						
**Africa**							
Morocco	FB_1_	3.5	8.9	6.9	6.4	5.2	[22]
	FB_2_	3.1	8	6.3	5.8	4.7	
Tunisia	DON	51	133	104	96	78.6	[33]
	15-ADON	1.7	4.5	3.5	3.2	2.6	
	HT-2	1.7	4.5	3.5	3.2	2.6	
	ZEN	1.7	4.5	3.5	3.2	2.6	
	ENB	52	134	104	96	78.6	
**America**							
Canada	OTA	1	2.6	2.1	1.9	1.5	[22]
**Asia**							
Iran	AFB_1_	4.5	11.6	9	8.4	6.8	[22]
Syria	OTA	0.16	0.42	0.33	0.30	0.25	[35]
**Europe**							
Italy	OTA	0.10	0.27	0.21	0.19	0.16	[5]
	NIV	34.4	88.8	69.1	63.9	52.2	
	FUS-X	253	653.4	508.5	470.3	383.7	
	DON	177.2	457.5	356.1	329.3	268.7	
	HT-2	21.8	56.4	43.9	40.6	33.1	
	β-ZEL	4.3	11.2	8.7	8	6.6	
	ENB	174.9	451.7	351.6	325.2	265.3	
	ENB_1_	13.5	34.8	27.1	25	20.4	
	ENB_4_	65.8	169.8	132.2	122.2	99.7	
	ENA_1_	11.4	29.3	22.8	21.1	17.2	
	BEA	2.4	5.3	4.1	3.8	3.1	
Portugal	PAT	4.2	10.4	8.1	7.5	6.1	[22]
	OTA	0.11	0.27	0.21	0.20	0.16	
Spain	AFB_1_	0.16	0.40	0.31	0.29	0.24	
	AFB_2_	0.02	0.04	0.03	0.03	0.03	
	AFG_1_	0.03	0.09	0.07	0.06	0.05	
	AFG_2_	0.01	0.02	0.01	0.021	0.01	
Spain	DON	202	521.8	406	375.6	306.4	[12]
Turkey	OTA	0.24	0.62	0.49	0.45	0.37	[22]

* Average consumption of cereal-based children’s products [42].

**Table 11:** Estimated daily intake of mycotoxins through the consumption of fruit products.

**Mycotoxin**	**Product**	**Origin**	**EDI (ng/kg/day)**						**Reference**
			**Age (months)**	4–5	5–6	6–7	7–8	8–9	
			**Weight (kg)**	6.95	7.4	7.78	8.1	8.4	
			Consumption * (g/day)	80	75	120	120	120	
			Consumption ** (g/day)	-	75	120	120	120	
**OTA**	**Solid** **fruit products**	**Asia**							
		Syria	Peach and apple puree	1.1	1	1.5	1.5	1.4	[35]
			Fruit cocktail puree	1	0.9	1.3	1.3	1.2	
**PAT**	**Solid** **fruit products**	**Asia**							
		Qatar	Apple compote	12.6	11.1	16.8	16.2	15.6	[41]
		**Europe**							
		Spain	Apple compote	85.18	75	114	109.6	105.7	[37]
			Multifruit compote	79.4	69.9	106.4	102.2	98.6	
		Italy	Puree and fruit compote	72.3	63.6	96.9	93	89.7	[38]
		Serbia	Apple puree	33.4	29.4	44.7	43	41.4	[40]
			Multi-fruit puree	40.3	35.5	54	51.8	50	
	**Juice** **products**	**Asia**							
		China	Apple juice		94.3	143.4	137.8	132.8	[39]
		Qatar	Apple juice		31.1	47.3	45.4	43.8	[41]
		**Europe**							
		Spain	Apple juice		76	115.7	111.1	107.1	[37]

* Average consumption of semi-solid fruit products [42]; ** Average consumption of liquid fruit products [42].

**Table 12:** Risk evaluation of OTA in breast milk.

Origin	TDI%									TDI (ng/kg b.w/day)	Reference
	0–1 Month	1–2 Months	2–3 Months	3–4 Months	4–5 Months	5–6 Months	6–7 Months	7–8 Months	8–9 Months		
**Africa**										14	
Egypt	25	21	18	18	17	17	15	14	16		[4]
**America**											
Brazil	5	4	3	3	3	3	3	3	3		[21]
Chile	94	79	66	68	64	63	58	54	60		
**Asia**											
Iran	1194	1013	837	870	815	806	743	691	762		[21]
**Europe**											
Italy	24	20	17	17	16	16	15	14	15		[20] [21]
Turkey	638	541	447	465	435	430	397	369	407		[21]

**Table 13:** Risk evaluation of mycotoxins in infant formulas.

Mycotoxin	Origin	TDI%									TDI (ng/kg b.w/day)	Reference
		0–1 Month	1–2 Months	2–3 Months	3–4 Months	4–5 Months	5–6 Months	6–7 Months	7–8 Months	8–9 Months		
**NIV**	**Asia**										1200	
	South Korea	6	6	6	5	6	5	4	4	3		[28]
**OTA**	**Africa**										14	
	Burkina Faso	15	16	16	14	15	14	11	11	8		[21]
	**America**											
	Canada	22	23	23	21	22	21	16	15	11		[21]
	**Europe**											
	Italy	13	14	14	13	14	13	10	9	7		[21]
	Turkey	3	3	3	3	3	3	2	2	1		
**ZEN**	**Europe**										250	
	Italy	0.22	0.23	0.23	0.21	0.23	0.21	0.16	0.15	0.11		[31]

**Table 14:** Risk evaluation of mycotoxins in cereal-based products.

Origin	Mycotoxin	TDI%					TDI (ng/kg b.w/day)	Reference
		4–5 Months	5–6 Months	6–7 Months	7–8 Months	8–9 Months		
**Africa**								
Morocco	FB_1_	0.17	0.45	0.35	0.32	0.26	2000	[21]
	FB_2_	0.16	0.40	0.31	0.29	0.24	2000	
Tunisia	DON	5	13	10	10	8	1000	[33]
	15-ADON	0.17	0.45	0.35	0.32	0.26	1000	
	HT-2	2	4	3	3	3	100	
	ZEN	0.69	1.78	1.39	1.28	1.05	250	
**America**								
Canada	OTA	7	19	15	13	11	14	[21]
**Asia**								
Syria	OTA	1	3	2	2	2	14	[35]
**Europe**								
Italy	OTA	0.74	2	1.5	1	1	14	[5]
	NIV	3	7	6	5	4	1200	
	DON	18	46	36	33	27	1000	
	HT-2	22	56	44	41	33	100	
	β-ZEL	1.73	4.5	3.5	3	3	250	
Portugal	PAT	1	3	2	2	1.5	400	[21]
	OTA	0.75	1.94	1.51	1.40	1.14	14	
Spain	DON	20	52	41	38	31	1000	[12]
Turkey	OTA	2	4.5	3.5	3	3	14	[21]

**Table 15:** Risk evaluation of mycotoxins in fruit products (puree, compote and juice).

Mycotoxin	Origin	Food	TDI%					TDI (ng/kg b.w/day)	Reference
			4–5 Months	5–6 Months	6–7 Months	7–8 Months	8–9 Months		
**OTA**	**Asia**							14	
	Syria	Peach and apple puree	8	7	11	10	10		[35]
		Fruit cocktail puree	7	6	10	9	9		
**PAT**	**Asia**							400	
	Qatar	Apple compote	3	3	4	4	4		[41]
	**Europe**								
	Spain	Apple compote	21	19	28	27	26		[37]
		Multifruit compote	20	17	27	26	25		
	Italy	Puree and fruit compote	18	16	24	23	22		[38]
	Serbia	Apple puree	8	7	11	11	10		[40]
		Multi-fruit puree	10	9	13	13	12		
	**Asia**								
	China	Apple juice		24	36	34	33		[39]
	Qatar	Apple juice		8	12	11	11		[41]
	**Europe**								
	Spain	Apple juice		19	29	28	27		[37]
	Serbia	Fruit juice		9	14	13	13		[40]

## Data Availability

The data presented in this study are available within these article.
